# Postprandial Glucose Variability Following Typical Meals in Youth Living with Type 1 Diabetes

**DOI:** 10.3390/nu16010162

**Published:** 2024-01-04

**Authors:** Susana R. Patton, Simon Bergford, Jennifer L. Sherr, Robin L. Gal, Peter Calhoun, Mark A. Clements, Michael C. Riddell, Corby K. Martin

**Affiliations:** 1 Nemours Children’s Health, Jacksonville, FL 32207, USA; 2 Jaeb Center for Health Research, Tampa, FL 33647, USA; 3 Yale School of Medicine, New Haven, CT 06510, USA; 4 Children’s Mercy Hospital, Kansas City, MO 64108, USA; 5 Muscle Health Research Centre, School of Kinesiology and Health Science, York University, Toronto, ON M3J1P3, Canada; 6 Pennington Biomedical Research Center, Louisiana State University, Baton Rouge, LA 70803, USA

**Keywords:** type 1 diabetes, pediatric diabetes, variability, glucose control, macronutrients

## Abstract

We explored the association between macronutrient intake and postprandial glucose variability in a large sample of youth living with T1D and consuming free-living meals. In the Type 1 Diabetes Exercise Initiative Pediatric (T1DEXIP) Study, youth took photographs before and after their meals on 3 days during a 10 day observation period. We used the remote food photograph method to obtain the macronutrient content of youth’s meals. We also collected physical activity, continuous glucose monitoring, and insulin use data. We measured glycemic variability using standard deviation (SD) and coefficient of variation (CV) of glucose for up to 3 h after meals. Our sample included 208 youth with T1D (mean age: 14 ± 2 years, mean HbA1c: 54 ± 14.2 mmol/mol [7.1 ± 1.3%]; 40% female). We observed greater postprandial glycemic variability (SD and CV) following meals with more carbohydrates. In contrast, we observed less postprandial variability following meals with more fat (SD and CV) and protein (SD only) after adjusting for carbohydrates. Insulin modality, exercise after meals, and exercise intensity did not influence associations between macronutrients and postprandial glycemic variability. To reduce postprandial glycemic variability in youth with T1D, clinicians should encourage diversified macronutrient meal content, with a goal to approximate dietary guidelines for suggested carbohydrate intake.

## 1. Introduction

In youth living with type 1 diabetes (T1D), the goal of daily therapy is to mimic near-normal glucose metabolism and minimize the occurrence of hyperglycemic and hypoglycemic events [1]. This is because research has linked excessive time spent in hyperglycemia to the development of long-term micro- and macrovascular complications [2]. Plus, there is evidence that greater time spent in hyperglycemia and hypoglycemia can increase the risk of acute complications including diabetic ketoacidosis (DKA) and severe hypoglycemia [3]. A key contributor to time spent above and below range is glycemic variability. Youth with T1D are vulnerable to periods of glycemic variability following meals and intensive physical activity, especially when these occur spontaneously [4]. Additionally, there is emerging evidence that the type of foods youth consume may contribute to postprandial glycemic variability. For instance, in a small randomized cross-over trial that included four test meals containing the same carbohydrate amount, Smart et al. [5] found youth with T1D experienced greater postprandial variability following the meal that was high in both protein and fat compared to the meal that was low in protein and fat. Moreover, a recent small clinical trial reported greater glycemic variability following test meals containing 50% of total daily energy from carbohydrates compared to 30% of total daily energy from carbohydrates [6]. In adolescents with T1D, researchers have yet to examine how the macronutrient content of free-living meals might relate to youths’ postprandial glycemic variability. Thus, using data from the Type 1 Diabetes Exercise Initiative Pediatric (T1DEXIP) study, we examined how the macronutrient content of typical meals influenced postprandial glycemic variability for youth living with T1D. Consistent with published trial results, we hypothesized greater glycemic variability following free-living meals comprised of larger carbohydrate, fat, and protein content. In subgroup analyses, we also examined the influence of insulin modality, exercise following meals, and exercise intensity on the relationship between the macronutrient content of meals and youth’s postprandial glycemic variability.

## 2. Materials and Methods

The T1DEXIP study was a large observational study that recruited youth living with T1D from across the United States. Eligible youth were between 12 and 17 years old; diagnosed with T1D for at least three months; following an intensive insulin regimen for at least one month; and reporting a Physical Activity Questionnaire for Children and Adolescents (PAQ) survey [7] score of at least 1.5, indicating a moderate or high level of physical activity. We excluded youth reporting two or more severe hypoglycemic events in the past year and youth reporting three or more overnight hospitalizations for blood glucose control in the past year. The T1DEXIP study was approved by the Jaeb Center for Health Research Institutional Review Board (protocol code T1DEXIP and date of approval: 22 April 2021).

Using online surveys, youth self-reported on their insulin regimen, most recent HbA1c, and history of severe hypoglycemia and DKA events. We then trained youth via video conferencing to use a wrist-worn activity tracker (Garmin vivosmart^®^ 4, Garmin International Inc., Olathe, KS, USA) and a study-specific version of the Bant Diabetes smart phone application (University Health Network and the Hospital for Sick Children, Toronto, ON, Canada) to self-report activity type, duration, and perceived intensity of any physical activity events that lasted greater than 10 min. We also trained youth to use the Bant app to enter meal information (and insulin for multiple daily injection (MDI) users) and to take before and after meal pictures to record their food intake for any three days (including at least one weekend day) during the observation period. To minimize missing data, participants set reminders within the Bant app to receive prompts on the days they planned to collect food photos at their typical mealtimes for those days.

Youth participated in the study observation period for approximately 10 days. During this period, youth continued their usual schedule and engaged in typical forms of physical activity and exercise and consumed their usual meals, while we collected continuous glucose monitoring (CGM) data. Youth who were current Dexcom G6, (Dexcom, Inc., San Diego, CA, USA) users continued to use their personal CGM during the study period, while youth who did not use the Dexcom G6 used a blinded Dexcom G6 pro sensor. We also collected insulin pump data, when available.

The Ingestive Behavior Laboratory at the Pennington Biomedical Research Center analyzed food photos from the participants using the validated Remote Food Photography Method (RFPM) [8,9]. RFPM uses a trained human rater and specialized computer program to estimate the portion sizes of foods in the photos and link these to macronutrient data from the Food and Nutrient Database for Dietary Studies [10] and other online resources. Research shows RFPM to be accurate in assessing energy and other nutrient intake in adults compared to the gold standard [8] and has been used previously in youth with T1D [11].

### Statistical Methods

We calculated glucose standard deviation (SD) and glucose coefficient of variation (CV) to measure postprandial variability. We used postprandial periods up to three hours following each meal and required at least two hours of CGM data in each postprandial period to calculate outcomes. We used grams of carbohydrates, grams of protein per kg, and grams of fat to assess whether there was an association with postprandial glucose SD and CV. We fit a repeated measures linear regression model for each independent variable (e.g., carbohydrate, fat, protein) adjusting for HbA1c, outcome (either SD or CV of glucose levels) in the 24 h prior to the meal, glucose at the start of the meal, insulin on board, grams of fiber, and grams of carbohydrates with an exchangeable correlation structure to handle the repeated meals. We used robust mixed-effects regression models if residuals were highly skewed. Subgroup analyses explored whether any subgroups affected the relationship between nutritional content and postprandial variability using the same regression model but adding a main effect and interaction between the subgroup factor and nutritional content factor to the model. We examined the following subgroup factors: age, sex, mealtime of day, insulin on board, glucose at start of meal, and exercise following a meal.

There was no imputation of missing data. All *p*-values were two-sided, and we controlled the false discovery rate using the two-stage Benjamini–Hochberg adaptive false discovery rate correction procedure. We performed all statistical tests using SAS software, version 9.4 (SAS Institute, Cary, NC, USA).

## 3. Results

The T1DEXIP study enrolled and observed youth between 6 October 2021 and 17 December 2022. This analysis cohort included 208 youth (mean ± SD age 14 ± 2 years, mean HbA1c 7.1 ± 1.3% (54 ± 14.2 mmol/mol), mean BMI percentile 61 ± 27%, mean T1D duration 5.4 ± 3.9 years). Forty percent of youth identified as female, 83% identified as non-Hispanic White, 7% as Hispanic, 5% as more than one race, 2% as Asian, 2% as unreported, and <1% as American Indian/Alaskan Native. In our cohort, 16% of youth used MDI, 27% used an open-loop insulin pump (Pump), and 57% used a hybrid closed-loop insulin pump (HCL). Almost all youth (>99%) reported personal CGM use at enrollment. With respect to total energy, youth consumed 50 ± 21% calories from carbohydrates, 36 ± 17% calories from fat, and a median (quartiles) of 13% (7%, 19%) from protein.

We summarize the macronutrient content of the 1980 meals included in the analyses in Table 1. In general, the youth’s meals consisted of a median (quartiles) of 44 g (24, 69) of carbohydrates, 16 g (7, 29) of fat, and 2.7 g (1.1, 5.1) of fiber. The median protein content of meals adjusted for an individual youth’s weight was 0.25 g/kg (0.08, 0.48). Mean glucose before meals was 144 ± 61 mg/dL (8.0 ± 3.4 mmol/L) (Table S1). The mean postprandial maximum glucose was 206 ± 68 mg/dL (11.4 ± 3.8 mmol/L), and the median time to peak glucose was 76 min (39, 131). Postprandial mean glucose was similar for the fat and protein groups. Mean postprandial glucose SD was 30 ± 17 mg/dL (1.7 ± 0.8 mmol/L), and mean CV was 19 ± 10%.

**Figure 1:**
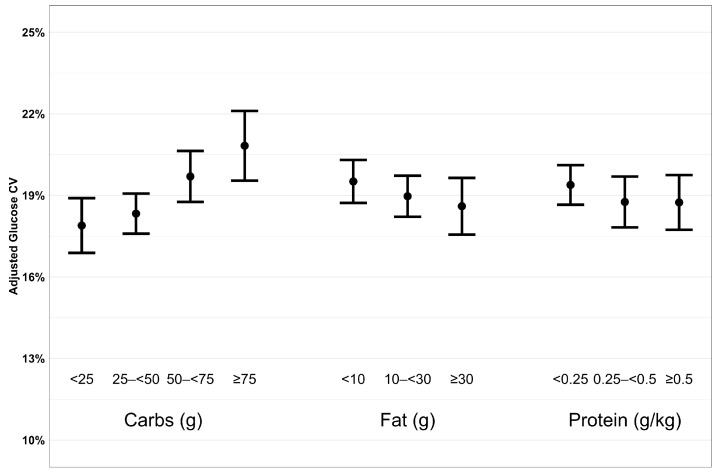
Plot of adjusted glucose CV by macronutrient group. Each plot has adjusted glucose CV and 95% confidence interval on the y-axis and macronutrient group on the x-axis.

**Figure 2:**
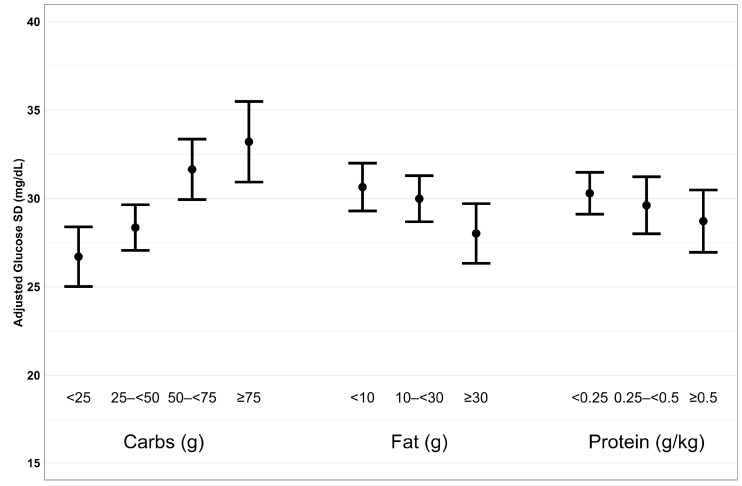
Plot of adjusted glucose SD by macronutrient group. Each plot has adjusted glucose SD and 95% confidence interval on the y-axis and macronutrient on the x-axis.

Consistent with our hypothesis, we observed greater postprandial glycemic variability following meals with more carbohydrates for both glucose CV and glucose SD (Figure 1 and Figure 2). Compared to meals containing <25 g of carbohydrates, the adjusted mean difference in glucose CV was 0.3% (95% confidence interval (CI): −0.8% to 1.5%) higher following meals containing 25 to <50 g of carbohydrates, 1.9% (CI: 0.6% to 3.2%) higher for meals with 50 to <75 g of carbohydrates, and 3.0% (CI: 1.4% to 4.6%) higher following meals containing ≥75 g of carbohydrates (*p* = 0.002 for overall effect of carbohydrates on glucose CV, Table 2). We observed similar results for SD, as meals with more carbohydrates had higher postprandial SD. Contrary to our hypothesis, we observed less postprandial glycemic variability following meals higher in fat content after adjusting for carbohydrates. Specifically, compared to meals containing <10 g of fat, the adjusted mean difference in glucose CV for meals containing 10 to <30 g and ≥30 g of fat was −0.5% (CI: −1.5% to 0.5%) and −1.0% (CI: −2.4% to 0.3%), respectively (*p* = 0.006). We observed similar results for SD as meals with more fat had lower postprandial SD. Also, contrary to our hypothesis, we observed less postprandial glucose SD for meals with higher protein content after adjusting for carbohydrates. Compared to meals containing <0.25 g/kg of protein, the adjusted mean differences in glucose SD for meals containing 0.25 to <0.50 g/kg and ≥0.50 g/kg of protein were −0.7 mg/dL (CI: −2.6 to 1.2 mg/dL) (−0.03 mmol/L [CI: −0.14 to 0.07 mmol/L]) and −1.8 mg/dL (CI: −3.8 to 0.3 mg/dL) (−0.1 mmol/L [CI: −0.2 to 0.02 mmol/L]), respectively (*p* = 0.03). Higher protein content did not have a significant effect on postprandial glucose CV.

There were no differences found in postprandial variability when models included insulin modality (Table S2), exercise following meals (Table S3), or intensity of exercise following meals (Table S4) as independent variables.

## 4. Discussion

Leveraging data from the T1DEXIP observational study provided a unique opportunity to assess the impact of macronutrient content on postprandial glycemic variability outside of rigorously structured clinical research trials in youth living with T1D while they continued their usual dietary and physical activity patterns. This assessment of data from the real world supported the hypothesis that greater carbohydrate content of meals relates to increased postprandial glycemic variability in youth with T1D. Surprisingly, there was an association between reduced postprandial glycemic variability and meals with higher fat content after adjusting for grams of carbohydrates in the meal. Also, meals with higher protein content had reduced postprandial glucose SD, but not glucose CV. Since SD is correlated with mean glucose, these findings suggest that higher protein intake may only be reducing the postprandial mean glucose with little effect on postprandial variability.

The T1DEXIP study collected detailed data on physical activity events of study participants as well as insulin delivery data. Due to this, we were able to explore factors related to treatment, such as insulin delivery modality, as well as event-level factors, like exercise during the postprandial period and intensity of exercise, in a subgroup analysis. Interestingly, we found no significant impact of insulin modality, exercise following meals, or intensity of exercise following meals on the associations between the carbohydrate, fat, and protein content of youth’s meals and their immediate postprandial glycemic variability.

Though there are a few published trials showing associations between greater carbohydrate, fat, and protein content of meals and greater postprandial glycemic variability in youth with T1D [5,6], these trials used small samples and standardized meals, which may limit their generalizability and clinical application. Our analyses of the T1DEXIP study data provide a unique opportunity to examine these associations using real-world data. Our models also control for multiple factors likely to affect postprandial glycemic variability in youth (e.g., youth’s HbA1c, glucose level at the start of the meal, insulin on board, grams of fiber, grams of carbohydrates, and either SD or CV of glucose levels in the 24 h prior to meals), which may enhance the scientific rigor of our findings. Our results specific to carbohydrate content are generally consistent with the published trial data showing greater postprandial glycemic variability following standardized meals that were higher in carbohydrate content [5,6]. Moreover, our results echo findings from a small observational study among very young children with T1D wherein researchers found greater glycemic variability following free-living meals that were higher in carbohydrate content [12].

In contrast, our results pertaining to the fat and protein content of meals deviate from published trial results. That is, in a previous randomized controlled trial, Smart et al. [5] observed greater postprandial glycemic variability up to five hours following standardized meals comprised of the same carbohydrate content and either higher fat or protein content. Additionally, they found the effect of fat and protein was cumulative, such that the standardized meal that was higher in both fat and protein associated with significantly greater postprandial glycemic variability compared to the standardized meals that were higher in fat or protein. In our models, we found reduced glycemic variability following meals with higher fat content after adjusting for carbohydrate content, and we found reduced glycemic variability following meals with higher protein content after adjusting for carbohydrate content. Our results align better with two recent studies that have also examined postprandial glycemic variability following free-living meals. For instance, like our results, Monzon et al. [12], found reduced glycemic variability following free-living meals with higher protein content in very young children with T1D, though they did not see an effect on glycemic variability following meals higher in fat content. Our results are also consistent with a small trial that examined the order of intake of carbohydrates, protein, and fat on postprandial variability in youth with T1D [13]. It is generally held that the addition of fat and protein to a fixed amount of carbohydrate in each meal delays the postprandial peak in glucose and extends glycemic excursions [14]. Thus, to reconcile differences observed in the glycemic patterns of youth with T1D following standardized versus free-living meals that were higher in either fat or protein, we offer a few considerations. First, the purpose of the study. In contrast to the previous controlled trial [5] that sought to examine glycemic impact of added fat or protein to a fixed amount of carbohydrate in a prescriptive fashion, we investigated the influence of macronutrient amount on glycemic variation in the real world. Second, the observation window. The previous controlled trial [5] measured glycemic variability up to five hours following the standardized meal, while our study and the two previously described studies [12,13] that used free-living meals measured glycemic variability up to three hours following the meal. It is possible that use of the shorter observation windows created an artificial ceiling on youth’s glycemic variability following meals. Third, the standardized meal. The previous controlled trial [5] only measured glycemic variability following breakfast, while our study assessed glycemic variability following all meals. It is possible time of day or bolus insulin taken for any snacks youth consumed within three hours of the observed meals could have reduced their glycemic variability. Finally, our study included a slight majority of youth (57%) on HCL, while the previous controlled trial [5] did not. As such, it is possible we observed less glycemic variability following meals higher in either fat or protein because the algorithm underlying youth’s HCL systems were automatically adjusting the youth’s insulin dose to correct for any projected deviation from target range. Of note, there are data in persons with type 2 diabetes suggesting different glycemic effects associated with the source of protein (i.e., plant versus animal) in their diet [15]. It does not seem this association has received much attention in persons with T1D. Therefore, a future study may want to investigate this association more in depth in youth with T1D.

As noted earlier, youth with T1D are vulnerable to developing hypoglycemia and/or hyperglycemia with planned and spontaneous physical activity [4]. As a result, expert guidelines exist to help youth with T1D consume adequate grams of carbohydrates before exercise to reduce their risk of hypoglycemia [16]. However, less is known about how exercise and the macronutrient content of meals may relate to postprandial glycemic variability in youth with T1D. To our knowledge, we are the first to examine these associations based on the carbohydrate and fat content of youth’s meals. We were surprised not to observe an effect of exercise during the postprandial period on glycemic variability following meals higher in carbohydrate content. Clinically, this result suggests that to reduce postprandial glycemic variability in youth with T1D, it may be more effective to target their diet than to prescribe physical activity, though physical activity has other health benefits and should not be discounted altogether [4,16]. One recent study examined the effect of exercise on the association between protein and glycemia in youth with T1D [17]. This study used data from free-living meals and youth-reported episodes of physical activity collected from 112 adolescents with T1D participating in a randomized clinical trial testing an adaptive behavioral intervention. Researchers found daily protein intake ≥1.2 g/kg associated with greater time in range and less time above range for the 24 h following exercise. However, when examining for specific effects of post-exercise protein intake on post-exercise glycemia, they only found associations for female adolescents, namely, every 0.25 g/kg of post-exercise protein intake relating to 1.4% less time below range and 2.3% time within range [17]. Our model was slightly different because we examined protein intake in meals occurring before exercise versus after exercise. We also used different measures of variability and applied a narrower observation window when examining glycemic variability (3 h versus 24 h post-exercise). It is possible that to observe any exercise effect on the association between the protein content of meals and postprandial glycemic variability, a longer observation window is required.

Our results associating greater glycemic variability following meals higher in carbohydrates in youth with T1D underscore the importance of teaching healthy eating principles as part of clinical nutritional counseling. Meals that include whole grains, legumes, vegetables, and fruits offer many healthful vitamins and minerals and may help to reduce postprandial glycemic excursions [18]. There is also evidence suggesting early administration of mealtime insulin ≈ 15–20 min before the meal can be an effective strategy to minimize postprandial glycemic variability [19,20]. Per international guidelines, data do not currently support recommending very low carbohydrate diets (<20–50 g/day) for youth with T1D, and the findings related to carbohydrate restriction are mixed [18]. Here, again, it may be advisable for providers to counsel families regarding healthy eating principles, how to replace higher glycemic index (GI) carbohydrates with lower GI choices, and preprandial insulin administration when addressing glycemic variability. Whilst the addition of fat or protein to meals can help to delay glucose absorption [21,22,23] and our data suggest reduced glycemic variability following free-living meals higher in either fat or protein in youth with T1D, we encourage providers to continue to recommend moderate fat and saturated fat intake because of the associated risk of cardiovascular disease [18,24]. Regarding protein intake, we encourage youth with T1D to obtain personalized recommendations from a dietician to ensure a daily intake level that will support normal growth and physical activity.

As a limitation, we note that our study design was observational and therefore we cannot assume any causation when relating youth’s macronutrient intake to their post-prandial glycemic variability. We acknowledge that our data focused on free-living meals without any standardization. With self-reported data, there is a risk of missing data if youth did not record all meals or snacks. However, we limited food photo collection to three days to reduce data reporting fatigue.

It is possible that there are other confounders not assessed that could have impacted the macronutrient effect on postprandial variability, though we did design our models to adjust for many known factors likely to influence postprandial glycemia. Related, we acknowledge that dietary impacts on postprandial glycemia can be individualized. Thus, to address this, our analyses mainly assessed macronutrients and postprandial glycemia within participants versus between participants. While protein intake was scaled for body mass in our study, which is customary for growing youth [25], carbohydrate and fat intake were not. We also acknowledge that we only included grams of fiber as a potential confounder in our models versus examining for any glycemic effects of fiber per se. Finally, we acknowledge that the T1DEXIP study recruited a cohort of youth with T1D who were already engaged in at least moderate levels of daily physical activity and had a mean HbA1c of 7.1%. It is possible these cohort characteristics could make our results less generalizable to typical youth living with T1D. Yet, as a counterpoint, our study cohort consumed a diet comparable to other published samples [26,27,28] and close to international guidelines [18] for percent calories from carbohydrate, fat, and protein, which may provide evidence of generalizability.

As strengths of our study, we think it is important to note the large sample size for the T1DEXIP study, its use of multiple technologies and often passive data collection methods (e.g., CGM, activity wearables), and its use of RFPM to collect and analyze the youth’s dietary intake. Previous trials have shown a tendency for people to under-report food intake [29] and over-report physical activity [30]. Thus, we believe these more objective and, in some cases, passive data collection methods improve the rigor of our data, though we also acknowledge these methods maintain a risk of data loss due to technological disruptions, inaccuracy if the activity wearable is not worn correctly, and participant reactivity [31]. Because the T1DEXIP study only collected free-living data, it is possible findings may be more generalizable than in laboratory settings. Finally, we identify our inclusion of multiple dietary and T1D-specific variables related to postprandial glycemic variability in our models as a strength.

## 5. Conclusions

When exploring how meals of different macronutrient content relate to postprandial glucose variability in youth with T1D, most of the existing studies have been small and used standardized meals [5,6]. Thus, we extend the literature exploring these associations in a large sample of youth consuming their normal nutrient intake during this observational study. Overall, our results suggest greater glycemic variability following meals higher in carbohydrate content, while we observed lower variability following meals higher in fat or protein. Notably, in our sub-analyses, insulin modality, exercise in the postprandial period, and exercise intensity did not appear to influence the relationship between mealtime macronutrients and postprandial glycemic variability. A future direction may be to conduct another large observational study of free-living meals in youth with T1D, this time extending the postprandial window to up to five hours to closer approximate controlled studies [5,6]. This study provides further evidence underscoring the potential impact of targeting diet and carbohydrate intake closely approximating the dietary guidelines [1,18] as two methods that may help to reduce postprandial glycemic variability in youth with T1D.

## Figures and Tables

**Table 1:** Meal characteristics. Data are median (quartiles).

		Carbohydrates				Fat			Protein		
	Overall	<25 g	25–<50 g	50–<75 g	≥75 g	<10 g	10–<30 g	≥30 g	<0.25 g/kg	0.25–<0.50 g/kg	≥0.5 g/kg
Number of Meals	1980	507	603	458	412	695	815	470	1000	514	466
Carbohydrates (g)	44 (24, 69)	16 (11, 19)	37 (30, 43)	61 (55, 67)	96 (83, 114)	24 (14, 43)	50 (33, 68)	72 (46, 99)	30 (18, 49)	57 (35, 80)	68 (44, 97)
Protein (g/kg)	0.25	0.05	0.20	0.31	0.49	0.06	0.28	0.61	0.08	0.36	0.70
	(0.08, 0.48)	(0.02, 0.19)	(0.08, 0.40)	(0.17, 0.51)	(0.33, 0.72)	(0.02, 0.16)	(0.14, 0.45)	(0.42, 0.83)	(0.03, 0.16)	(0.31, 0.43)	(0.59, 0.88)
Fat (g)	16 (7, 29)	6 (1, 11)	14 (8, 23)	20 (11, 30)	32 (20, 47)	4 (1, 7)	18 (14, 23)	42 (35, 53)	8 (3, 14)	21 (14, 31)	36 (24, 51)
Fiber (g)	2.7	0.7	2.2	3.7	6.2	1.0	3.1	5.2	1.5	3.7	5.3
	(1.1, 5.1)	(0.1, 1.6)	(1.2, 3.5)	(2.2, 5.5)	(4.3, 8.8)	(0.3, 2.7)	(1.6, 5.1)	(3.1, 7.7)	(0.5, 3.1)	(2.0, 5.6)	(2.9, 7.6)
Meal weight (g)	248	68	215	305	481	124	246	400	121	307	427
	(111, 401)	(28, 161)	(118, 327)	(208, 419)	(358, 661)	(32, 272)	(135, 393)	(285, 585)	(49, 248)	(204, 430)	(327, 629)
Insulin on board (U/kg)	0.07	0.05	0.07	0.08	0.09	0.06	0.07	0.07	0.06	0.07	0.08
	(0.02, 0.12)	(0.02, 0.09)	(0.02, 0.11)	(0.02, 0.14)	(0.03, 0.15)	(0.02, 0.11)	(0.02, 0.13)	(0.02, 0.13)	(0.02, 0.12)	(0.02, 0.13)	(0.03, 0.13)

**Table 2:** Effect of macronutrients on postprandial glycemic variability.

	N	Mean ± SD	Adjusted Mean Difference (95% CI)	*p*-Value ^a^
Glucose CV (%)				
Overall	1980	19 ± 10%	-	-
Carbohydrates				0.002
<25 g	507	18 ± 9%	Reference	
25 to <50 g	603	18 ± 9%	0.3% (−0.8%, 1.5%)	
50 to <75 g	458	20 ± 10%	1.9% (0.6%, 3.2%)	
≥75 g	412	21 ± 10%	3.0% (1.4%, 4.6%)	
Fat				0.006
<10 g	695	19 ± 9%	Reference	
10 to <30 g	815	19 ± 10%	−0.5% (−1.5%, 0.5%)	
≥30 g	470	19 ± 10%	−1.0% (−2.4%, 0.3%)	
Protein				0.19
<0.25 g/kg	1000	19 ± 9%	Reference	
0.25 to <0.50 g/kg	514	19 ± 9%	−0.7% (−1.8%, 0.4%)	
≥0.50 g/kg	466	19 ± 10%	−0.9% (−2.1%, 0.3%)	
Glucose SD (mg/dL)				
Overall	1980	30 ± 17	-	-
Carbohydrates				<0.001
<25 g	507	26 ± 15	Reference	
25 to <50 g	603	28 ± 15	1.6 (−0.4, 3.6)	
50 to <75 g	458	32 ± 18	5.1 (2.8, 7.4)	
≥75 g	412	33 ± 19	6.6 (3.8, 9.3)	
Fat				<0.001
<10 g	695	29 ± 17	Reference	
10 to <30 g	815	30 ± 17	−0.5 (−2.3, 1.3)	
≥30 g	470	30 ± 17	−2.7 (−5.0, −0.4)	
Protein				0.03
<0.25 g/kg	1000	29 ± 16	Reference	
0.25 to <0.50 g/kg	514	30 ± 17	−0.7 (−2.6, 1.2)	
≥0.50 g/kg	466	30 ± 18	−1.8 (−3.8, 0.3)	

^a^—*p*-value on the effect of nutritional content (carbohydrates, fat, or protein) on glucose CV and glucose SD based on a repeated measures linear regression model adjusting for HbA1c, outcome in 24 h prior to meal, glucose at the start of the meal, insulin on board, grams of fiber, and grams of carbohydrates with an exchangeable correlation structure.

## Data Availability

Deidentified data are available through Vivli: https://doi.org/10.25934/PR00008429 (accessed on 28 December 2023).

## Supplementary Materials

The following supporting information can be downloaded at https://www.mdpi.com/article/10.3390/nu16010162/s1, Table S1: Postprandial characteristics. Table S2: Effect of insulin modality and macronutrients on postprandial glycemic variability. Table S3: Effect of exercise following meals and macronutrients on postprandial glycemic variability. Table S4: Effect of exercise intensity and macronutrients on postprandial glycemic variability.
