# Supplementary material for: Postprandial Glucose Variability Following Typical Meals in Youth Living with Type 1 Diabetes
Source: Nutrients. 2024 Jan 4;16(1):162. doi: 10.3390/nu16010162 (PMC10781146; doi:10.3390/nu16010162)
Supplement: Supplementary file 1 [file nutrients-16-00162-s001.zip › nutrients-2719051-supplementary.pdf]

Supplemental Table S1. Postprandial Characteristics

		Carbohydrates				Fat			Protein		
	Overall	<25 g	25-<50 g	50-<75 g	≥75 g	<10 g	10-<30 (g)	≥30 g	<0.25 g/kg	0.25-<0.50 g/kg	≥0.50 g/kg
# of Meals	1980	507	603	458	412	695	815	470	1000	514	466
Glucose before Meal	144 ± 61	138 ± 62	144 ± 59	148 ± 61	148 ± 65	140 ± 63	147 ± 61	147 ± 59	141 ± 61	147 ± 62	149 ± 62
% TIR 70-180 mg/dL	75% (39%, 100%)	83% (48%, 100%)	72% (36%, 100%)	68% (31%, 95%)	72% (37%, 94%)	77% (39%, 100%)	72% (33%, 97%)	75% (44%, 100%)	75% (39%, 100%)	72% (31%, 97%)	75% (44%, 100%)
Mean Glucose (mg/dL)	160 ± 57	150 ± 55	163 ± 56	166 ± 59	160 ± 58	157 ± 59	164 ± 58	156 ± 51	158 ± 57	164 ± 59	159 ± 54
% Time >180 mg/dL	17% (0%, 61%)	0% (0%, 48%)	21% (0%, 64%)	25% (0%, 69%)	17% (0%, 58%)	11% (0%, 58%)	19% (0%, 66%)	17% (0%, 56%)	16% (0%, 58%)	20% (0%, 69%)	17% (0%, 56%)
% Meals with Hyper event >250 mg/dL	20%	16%	20%	25%	21%	21%	21%	19%	20%	22%	18%
% Meals with Hypo event <70 mg/dL	11%	13%	9%	10%	12%	13%	9%	10%	12%	10%	9%
% Meals with Hypo event <54 mg/dL	2%	1%	2%	3%	2%	2%	2%	2%	2%	2%	2%
Maximum glucose (mg/dL)	206 ± 68	190 ± 66	207 ± 65	215 ± 70	213 ± 71	201 ± 71	210 ± 68	204 ± 63	202 ± 68	212 ± 70	206 ± 66
Time to peak (min)	76 (39, 131)	75 (33, 127)	77 (44, 128)	78 (41, 136)	73 (38, 138)	75 (40, 127)	78 (41, 131)	73 (34, 137)	76 (40, 131)	76 (41, 131)	74 (36, 130)
Excursion (mg/dL)	52 (19, 92)	44 (12, 80)	52 (24, 92)	58 (21, 99)	50 (19, 96)	52 (20, 92)	54 (20, 98)	46 (16, 85)	52 (21, 92)	55 (20, 97)	47 (17, 87)

Data are Mean ± SD or Median (Quartiles) unless otherwise indicated.

Supplemental Table S2. Effect of Insulin Modality and Macronutrients on Postprandial Glycemic Variability

	MDI/Pump		Closed Loop		Adjusted Mean Difference (95% CI)	Interaction P-value [a]
	N	Mean ± SD	N	Mean ± SD		
**Glucose CV (%)**						
Carbohydrates						0.41
<25 g	215	16% ± 8%	292	19% ± 9%	-2.9% (-5.5%, -0.3%)	
25 to <50 g	250	17% ± 9%	353	19% ± 9%	-2.0% (-4.3%, 0.3%)	
50 to <75 g	181	19% ± 10%	277	20% ± 10%	-1.8% (-4.5%, 0.9%)	
≥75 g	175	21% ± 10%	237	21% ± 10%	-0.1% (-2.9%, 2.7%)	
Fat						0.32
<10 g	282	17% ± 9%	413	20% ± 9%	-2.2% (-4.3%, 0.0%)	
10 to <30 g	331	18% ± 9%	484	20% ± 10%	-1.8% (-3.9%, 0.2%)	
≥30 g	208	19% ± 10%	262	20% ± 10%	-1.0% (-3.7%, 1.6%)	
Protein						0.41
<0.25 g/kg	381	17% ± 9%	619	20% ± 10%	-2.2% (-4.0%, -0.3%)	
0.25 to <0.50 g/kg	228	18% ± 9%	286	20% ± 10%	-2.7% (-5.2%, -0.1%)	
≥0.50 g/kg	212	20% ± 10%	254	19% ± 10%	0.2% (-2.4%, 2.9%)	
**Glucose SD (mg/dL)**						
Carbohydrates						0.32
<25 g	215	23 ± 16	292	28 ± 14	-4.5 (-8.9, -0.0)	
25 to <50 g	250	26 ± 14	353	30 ± 16	-3.8 (-7.8, 0.3)	
50 to <75 g	181	32 ± 20	277	32 ± 17	-0.6 (-5.3, 4.0)	
≥75 g	175	32 ± 18	237	33 ± 19	0.2 (-4.7, 5.0)	
Fat						0.30
<10 g	282	26 ± 17	413	31 ± 17	-4.0 (-7.8, -0.2)	
10 to <30 g	331	29 ± 17	484	31 ± 16	-2.1 (-5.6, 1.3)	
≥30 g	208	29 ± 18	262	30 ± 17	-0.4 (-4.9, 4.2)	
Protein						0.41
<0.25 g/kg	381	26 ± 16	619	31 ± 17	-3.4 (-6.6, -0.3)	
0.25 to <0.50 g/kg	228	29 ± 17	286	32 ± 17	-3.3 (-7.6, 1.1)	
≥0.50 g/kg	212	30 ± 20	254	30 ± 16	0.9 (-3.6, 5.5)	

a - P-value on the effect of insulin modality (MDI/pump or AID) on the relationship between nutritional content and outcome (glucose CV or glucose SD) based on a repeated measures linear regression model adjusting for HbA1c, outcome in 24 hours prior to meal, glucose at the start of the meal, insulin on board, grams of fiber, grams of carbohydrates, and main effect of nutritional content (carbohydrates, fat, protein) and insulin modality with an exchangeable correlation structure

Supplemental Table S3. Effect of Exercise Following Meals and Macronutrients on Postprandial Glycemic Variability

	No Exercise		Exercise		Adjusted Mean Difference (95% CI)	Interaction P-value [a]
	N	Mean ± SD	N	Mean ± SD		
**Glucose CV (%)**						
Carbohydrates						0.21
<25 g	369	17% ± 8%	138	19% ± 10%	-3.4% (-6.3%, -0.6%)	
25 to <50 g	451	17% ± 9%	152	20% ± 10%	-2.6% (-5.3%, 0.0%)	
50 to <75 g	323	19% ± 10%	135	22% ± 10%	-2.8% (-5.7%, 0.1%)	
≥75 g	285	21% ± 11%	127	21% ± 10%	0.3% (-2.7%, 3.4%)	
Fat						0.41
<10 g	521	18% ± 9%	174	21% ± 10%	-2.8% (-5.3%, -0.4%)	
10 to <30 g	576	18% ± 9%	239	21% ± 9%	-3.0% (-5.2%, -0.8%)	
≥30 g	331	19% ± 10%	139	20% ± 9%	-0.3% (-3.2%, 2.6%)	
Protein						0.20
<0.25 g/kg	734	18% ± 9%	266	21% ± 10%	-3.0% (-5.0%, -0.9%)	
0.25 to <0.50 g/kg	380	19% ± 9%	134	21% ± 10%	-2.6% (-5.4%, 0.2%)	
≥0.50 g/kg	314	19% ± 10%	152	20% ± 9%	-0.6% (-3.4%, 2.2%)	
**Glucose SD (mg/dL)**						
Carbohydrates						0.15
<25 g	369	25 ± 14	138	29 ± 19	-5.4 (-10.4, -0.5)	
25 to <50 g	451	27 ± 15	152	31 ± 17	-3.8 (-8.4, 0.8)	
50 to <75 g	323	32 ± 18	135	33 ± 18	-3.3 (-8.4, 1.7)	
≥75 g	285	33 ± 20	127	32 ± 17	2.0 (-3.2, 7.2)	
Fat						0.30
<10 g	521	28 ± 16	174	31 ± 20	-4.3 (-8.6, -0.0)	
10 to <30 g	576	29 ± 16	239	32 ± 17	-3.6 (-7.4, 0.2)	
≥30 g	331	30 ± 18	139	29 ± 16	0.3 (-4.7, 5.2)	
Protein						0.15
<0.25 g/kg	734	28 ± 16	266	32 ± 18	-4.6 (-8.1, -1.1)	
0.25 to <0.50 g/kg	380	30 ± 17	134	32 ± 18	-2.8 (-7.7, 2.1)	
≥0.50 g/kg	314	30 ± 19	152	30 ± 17	0.3 (-4.6, 5.1)	

a - P-value on the effect of exercise after meal on the relationship between nutritional content and outcome (glucose CV or glucose SD) based on a repeated measures linear regression model adjusting for HbA1c, outcome in 24 hours prior to meal, glucose at the start of the meal, insulin on board, grams of fiber, grams of carbohydrates, and main effect of nutritional content (carbohydrates, fat, protein) and exercise status with an exchangeable correlation structure

Supplemental Table S4. Effect of Exercise Intensity and Macronutrients on Postprandial Glycemic Variability

	No Exercise		Low Intensity		Medium Intensity		High Intensity	
	**N**	**Mean ± SD**	**N**	**Mean ± SD**	**N**	**Mean ± SD**	**N**	**Mean ± SD**
**Glucose CV (%)**								
Carbohydrates								
<25 g	369	17% ± 8%	43	19% ± 11%	77	20% ± 10%	18	21% ± 10%
25 to <50 g	451	17% ± 9%	41	21% ± 10%	97	20% ± 9%	14	22% ± 9%
50 to <75 g	323	19% ± 10%	36	20% ± 9%	88	22% ± 10%	11	19% ± 7%
≥75 g	285	21% ± 11%	45	20% ± 7%	68	22% ± 10%	14	23% ± 15%
Fat								
<10 g	521	18% ± 9%	46	20% ± 11%	106	21% ± 10%	22	20% ± 8%
10 to <30 g	576	18% ± 9%	72	21% ± 9%	147	21% ± 9%	20	23% ± 12%
≥30 g	331	19% ± 10%	47	17% ± 8%	77	21% ± 10%	15	20% ± 11%
Protein								
<0.25 g/kg	734	18% ± 9%	78	20% ± 10%	162	21% ± 10%	26	20% ± 9%
0.25 to <0.50 g/kg	380	19% ± 9%	39	19% ± 9%	78	21% ± 10%	17	23% ± 13%
≥0.50 g/kg	314	19% ± 10%	48	19% ± 10%	90	20% ± 9%	14	20% ± 11%
**Glucose SD (mg/dL)**								
Carbohydrates								
<25 g	369	25 ± 14	43	28 ± 21	77	29 ± 17	18	32 ± 20
25 to <50 g	451	27 ± 15	41	36 ± 19	97	30 ± 16	14	28 ± 11
50 to <75 g	323	32 ± 18	36	32 ± 20	88	34 ± 18	11	28 ± 19
≥75 g	285	33 ± 20	45	31 ± 15	68	33 ± 18	14	29 ± 17
Fat								
<10 g	521	28 ± 16	46	33 ± 23	106	31 ± 19	22	29 ± 19
10 to <30 g	576	29 ± 16	72	35 ± 19	147	31 ± 16	20	32 ± 17
≥30 g	331	30 ± 18	47	26 ± 15	77	31 ± 17	15	26 ± 13
Protein								
<0.25 g/kg	734	28 ± 16	78	33 ± 19	162	31 ± 17	26	31 ± 20
0.25 to <0.50 g/kg	380	30 ± 17	39	31 ± 20	78	33 ± 18	17	30 ± 16
≥0.50 g/kg	314	30 ± 19	48	31 ± 18	90	30 ± 16	14	26 ± 12
